# The Fis Nucleoid Protein Negatively Regulates the Phase Variation *fimS* Switch of the Type 1 Pilus Operon in Enteropathogenic *Escherichia coli*

**DOI:** 10.3389/fmicb.2022.882563

**Published:** 2022-04-28

**Authors:** Zeus Saldaña-Ahuactzi, Jorge Soria-Bustos, Verónica I. Martínez-Santos, Jorge A. Yañez-Santos, Ygnacio Martínez-Laguna, María Lilia Cedillo-Ramirez, José L. Puente, Jorge A. Girón

**Affiliations:** ^1^Paul G. Allen School for Global Health, College of Veterinary Medicine, Washington State University, Pullman, WA, United States; ^2^Instituto de Ciencias de la Salud, Universidad Autónoma del Estado de Hidalgo, Pachuca, Mexico; ^3^CONACyt Facultad de Ciencias Químico-Biológicas, Universidad Autónoma de Guerrero, Chilpancingo, Mexico; ^4^Facultad de Estomatología, Benemérita Universidad Autónoma de Puebla, Puebla, Mexico; ^5^Centro de Investigaciones en Ciencias Microbiológicas, Benemérita Universidad Autónoma de Puebla, Puebla, Mexico; ^6^Centro de Detección Biomolecular, Benemérita Universidad Autónoma de Puebla, Puebla, Mexico; ^7^Instituto de Biotecnología, Universidad Nacional Autónoma de México, Cuernavaca, Mexico

**Keywords:** Fis, phase variation, *fimS* switch, type 1 pilus, EPEC

## Abstract

In *Escherichia coli* the expression of type 1 pili (T1P) is determined by the site-specific inversion of the *fimS* ON–OFF switch located immediately upstream of major fimbrial subunit gene *fimA*. Here we investigated the role of virulence (Ler, GrlR, and GrlA) and global regulators (H-NS, IHF, and Fis) in the regulation of the *fimS* switch in the human enteropathogenic *E. coli* (EPEC) O127:H6 strain E2348/69. This strain does not produce detectable T1P and PCR analysis of the *fimS* switch confirmed that it is locked in the OFF orientation. Among the regulator mutants analyzed, only the ∆*fis* mutant produced significantly high levels of T1P on its surface and yielded high titers of agglutination of guinea pig erythrocytes. Expression analysis of the *fimA*, *fimB*, and *fimE* promoters using *lacZ* transcriptional fusions indicated that only P*fimA* activity is enhanced in the absence of Fis. Collectively, these data demonstrate that Fis is a negative regulator of T1P expression in EPEC and suggest that it is required for the FimE-dependent inversion of the *fimS* switch from the ON-to-OFF direction. It is possible that a similar mechanism of T1P regulation exists in other intestinal and extra-intestinal pathogenic classes of *E. coli*.

## Introduction

Type 1 pili (T1P) are hair-like structures produced by *Escherichia coli* strains and other members of the *Enterobacteriaceae* (Werneburg and Thanassi, 2018). The clinical importance of T1P has been clearly demonstrated in the pathogenesis of uropathogenic *E. coli* (UPEC) strains that colonize and cause disease in the urinary tract (Welch et al., 2002; Flores-Mireles et al., 2015). In the bladder, T1P mediate bacterial attachment of UPEC to mannose-containing receptors present on epithelial cells. T1P are also important colonization factors of avian pathogenic *E. coli* strains that cause respiratory disease and adherent-invasive *E. coli* (AIEC) strains that cause inflammation of the colon (La Ragione et al., 2000; La Ragione and Woodward, 2002; Kaper et al., 2004; Martinez-Medina and Garcia-Gil, 2014; Yang et al., 2020). The role of T1P in the pathogenesis of enteropathogenic *E. coli* (EPEC), a cause of childhood diarrhea in the developing world, remains unclear. An early study in human volunteers fed with EPEC strain E2348/69 showed that antibodies against T1P are developed during infection suggesting that these pili are produced *in vivo* (Levine et al., 1978). However, the contribution of T1P to the adherence and colonization of the small bowel has not been studied. In contrast, T1P appear to play an important role in the interaction of BFP-negative atypical EPEC strains with abiotic surfaces favoring biofilm formation (Hernandes et al., 2013; Nascimento et al., 2014).

T1P cause mannose-sensitive agglutination of guinea pig and fowl erythrocytes (Abraham et al., 1985; Klemm, 1986; Spaulding et al., 2018). These pili are composed of a major repeating FimA 17 kDa-subunit that form a helical structure of about 0.5 to 2.0 μm in length and a diameter of 7 nm (Hahn et al., 2002; Puorger et al., 2011). At the tip of the filament sits the FimH adhesin protein, which forms a fibrillum structure responsible for the receptor mannose-binding specificity (Puorger et al., 2011). Regulation of fimbrial expression in *E. coli* is determined in general by regulatory genetic elements as well as by environmental signals (Roesch and Blomfield, 1998; Hung et al., 2001; Blomfield and van der Woude, 2007; Müller et al., 2009; De la Cruz et al., 2017; Matter et al., 2018; Ares et al., 2019). Determining what fimbriae are expressed at particular sites in the host is crucial for the microorganism for tissue colonization and survival against the immune system and to adapt to different hosts and environments. The chromosomal *fimAICDFGH* gene cluster encode the machinery for T1P assembly (Klemm, 1986; Remaut et al., 2006). *fimA* is transcriptionally regulated by a phase variation mechanism involving the inversion of a 314-bp chromosomal DNA segment (*fimS*) located immediately upstream of *fimA* (Abraham et al., 1985; Klemm, 1986). The *fimA* promoter is believed to reside within this invertible *fimS* element and directs transcription of the *fimAICDFGH* gene cluster when *fimS* is in the ON orientation promoting production of pili. In the alternate OFF orientation, no pili are produced (Freitag et al., 1985; Blomfield et al., 1991). Further, T1P phase variation is controlled by two recombinases encoded by *fimB* and *fimE*, located upstream of *fimA* (Klemm, 1986; Dorman and Higgins, 1987). Both recombinases bind to specific half-sites that flank, and overlap with, the left and right inverted repeats (IRL and IRR, respectively; Gally et al., 1996). FimE and FimB act in opposite ways such that FimE shows a strong preference for the *fimS* switch in the OFF orientation whereas FimB facilitates switching in both directions (Klemm, 1986; McClain et al., 1991, 1993; McCusker et al., 2008). In wild-type cells, FimE activity predominates over FimB and hence, most bacteria are non-piliated. *fimB* mutants retain FimE thus they are locked in the OFF orientation (Klemm, 1986; McClain et al., 1993; Gally et al., 1996).

Several other regulatory DNA-binding proteins also bind to and affect the inversion of the *fimS* switch including the integration host factor (IHF), the histone-like protein (H-NS), and the leucine-responsive global regulatory protein (Lrp; Schmid, 1990; Blomfield et al., 1993; Finkel and Johnson, 1993; Roesch and Blomfield, 1998; Bessaiah et al., 2022). In wild-type strains, normal expression of *fimA* requires IHF, whereas IHF mutants have expression of *fimA* locked either in the ON or OFF phase. IHF plays a dual role in controlling *fimA* expression as it is required both for inversion of the *fimA* control region and efficient expression from the *fimA* promoter and this protein was shown to bind with high affinity to two sites within the *fimS* invertible element (Dorman and Higgins, 1987; Blomfield et al., 1997). The DNA-binding protein Lrp is involved in transcriptional activation and repression of metabolic genes in *E. coli* and it binds to the *fimS* switch promoting phase variation (Blomfield et al., 1993; Gally et al., 1993; Calvo and Matthews, 1994). Lrp binds to two sites in or near the *fimS* switch, where it acts positively on DNA inversion. This protein alters the trajectory of the invertible element to enhance the formation of a synaptic complex for recombination. Interestingly, both binding sites for Lrp and for IHF are overlapped, suggesting a possible interaction between these two regulators (Blomfield et al., 1993; Gally et al., 1994; Kelly et al., 2006).

Fis, the factor for inversion stimulation, is an *E. coli* host factor required for *in vitro* DNA inversion (Johnson et al., 1986; Koch et al., 1988; Finkel and Johnson, 1993). Fis affects the expression of multiple genes by binding to promoters containing the degenerate sequence 5‘GNNC/TA/TNNA/TNNT/CG/ANNC3’, where N can be any base (Finkel and Johnson, 1993; Hengen et al., 1997; Schneider et al., 2001; Feldman et al., 2006). Fis alters the conformation of DNA through bending as well as through contact with the a-subunit of RNA polymerase (Thompson et al., 1988; Bokal et al., 1997; Dorman and Deighan, 2003). Fis participates in site-specific recombination events, such as in the excision of the lambda phage, and acts as an enhancer of site-specific DNA inversion (Gille et al., 1991). Fis is a key activator of exponential phase genes and a repressor of stationary phase genes (Ross et al., 1990; Ball et al., 1992; Xu and Jhonson, 1995; González-Gil et al., 1996; Kelly et al., 2004; Mallik et al., 2004; Lenz and Bassler, 2007; Duprey et al., 2014).

In EPEC, Fis activates transcription of *E. coli* type three secreted proteins genes (*espA, espB*, and *espD*), and the virulence regulator gene *ler*, and represses expression of the bundle-forming pilus gene (*bfpA*; Goldberg et al., 2001). Fis also acts as a negative regulator of curli in EPEC and Enterohemorrhagic *E. coli* (EHEC) O157:H7 (Saldaña et al., 2009). The present study was initiated to investigate the role of global and virulence factors on the expression of T1P in EPEC and to further understand how the ON–OFF phase variation switch works in this organism. The data support a role for Fis as a negative regulator in the *fimS* phase variation switch of the *fim* operon. This study advances our knowledge on the regulation of a pilus structure that is widely distributed among the *Enterobacteriaceae*.

## Results

### Comparative Analysis of the *fim* Operon of Different *E. coli* Pathotypes

The nucleotide sequence of the *fim* operon of representative prototypic strains of EPEC, UPEC, NMEC, AIEC, EHEC, EAEC, and ETEC was analyzed using the NCBI Multiple Sequence Alignment Viewer, version 1.21.0. This analysis shows that the *fim* operon is highly conserved among the *E. coli* pathotypes, ranking from 97% to 98% identity (Supplementary Figure 1). We then sought to determine the homology of the *fimS* invertible element if these *E. coli* pathotypes. The T-Coffee software was used to align the sequences. (Supplementary Figure 2). The analysis shows that the *fimS* switch is highly conserved among the *E. coli*.

### Fis is a Negative Regulator of T1P

**Figure 1:**
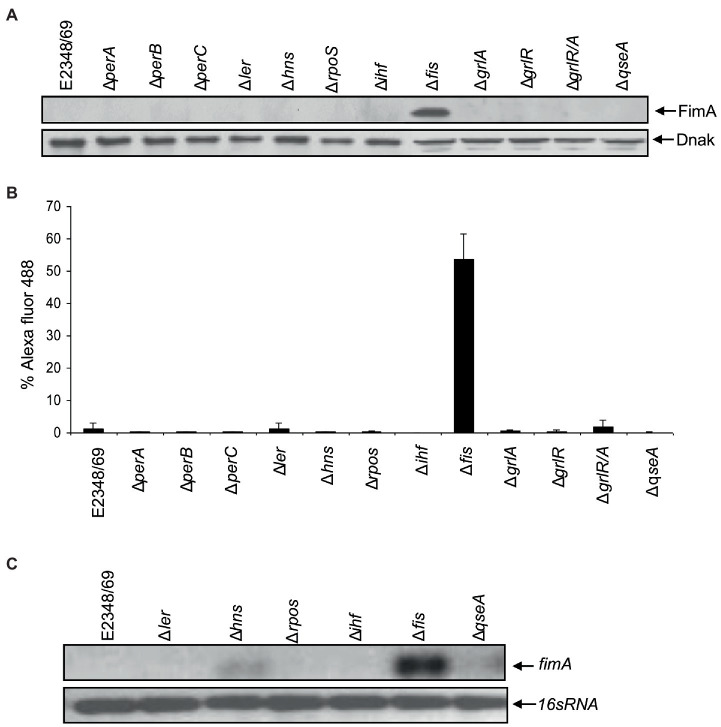
Fis is a negative regulator of T1P. **(A)** Western blot and **(B)** flow cytometry, using antibodies raised against T1P revealed that E2348/69 does not produce detectable amounts of T1P compared to the ∆*fis* mutant. **(C)** RT-PCR using primers for *fimA* gene.

**Figure 2:**
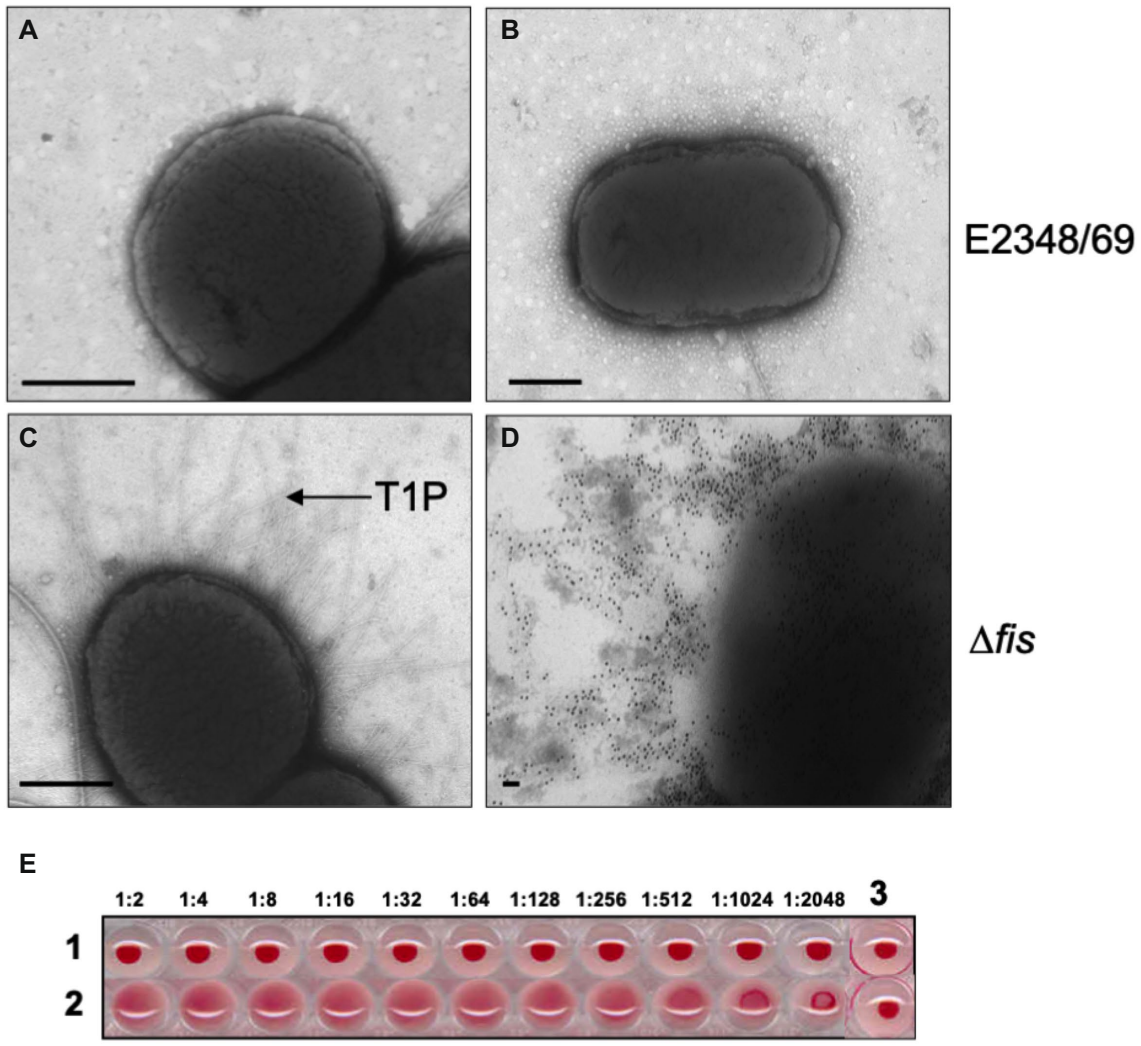
Transmission electron microscopy and hemagglutination. **(A,B)** Negative staining and immuno-staining of E2348/69 showing no production of T1P. **(C,D)** Negative staining and immuno-gold labeling of E2348/69Δ*fis* producing abundant T1P. Magnification bars, 0.5 μm. **(E)** Hemagglutinations (HA) showing (1) E2348/69, which does not produce T1P and consequently does not produce HA; (2) E2348/69Δ*fis* produces T1P and shows strong HA titer. (3) RBCs alone as negative control. The HA assay was done with 1% guinea pig red blood cells (RBCs) with the bacteria in the dilutions noted above.

Ultrastructural analysis of EPEC E2348/69 by electron microscopy has revealed that this strain does not produce detectable amounts of T1P when growing in Luria-Bertani (LB) broth at 37°C, suggesting that expression of T1P is under the influence of a strict regulatory control. A PCR-based analysis of the *fimS* switch of EPEC E2348/69 showed that this *fim* switch is locked in the OFF orientation. We set out to investigate the reason(s) for the lack of T1P in EPEC and so we began by studying the effect of mutations in virulence (*perABC, ler, grlA*, and *grlR*) and global (*hns, ihf, rpoS*, and *fis*) regulator genes in the production of T1P. These virulence and global regulators regulate the expression of EPEC chromosome- and plasmid-encoded virulence factors. Among all of the mutants analyzed, only the Δ*fis* mutant showed a dramatic effect on *fimA* expression. Namely, a significant increase of FimA synthesis was detected with anti-T1P antibodies by immunoblotting, a 50-fold increase in T1P production was recorded employing flow cytometry, and *fimA* expression by RT-PCR when compared to the wild-type strain and the other mutants (Figures 1A–C). Transmission electron microscopy analysis of the E2348/69Δ*fis* mutant (Figure 2C) confirmed these results, which showed an increased production of fimbriae on the surface of the bacteria as compared to E2348/69 (Figure 2A). Immunogold labeling with anti-T1P antibody confirmed the presence of T1P on the surface of E2348/69Δ*fis* (Figure 2D). No gold labeling was observed on E2348/69 (Figure 2B). To further confirm the identity of the pili structures produced by the Δ*fis* mutant, we purified the pili as described in Experimental Procedures. The pili purified from the E2348/69Δ*fis* mutant dissociated into 17 kDa protein subunits after HCl treatment in SDS-PAGE gels (Supplementary Figure 3) and the peptides generated after trypsin digestion were analyzed by mass spectrometry. The amino acid sequence of these peptides corresponded to the FimA amino acid (AATTVNGGTV) sequence of *E. coli* K-12 (data not shown). Given that T1P mediate mannose-sensitive hemagglutination (HA) of guinea pig red blood cells, we tested the ability of E2348/69Δ*fis* and the wild-type strain to hemagglutinate guinea pig erythrocytes in the presence and absence of 1% D-mannose. While the wild-type strain showed no HA reaction, the E2348/69Δ*fis* showed a high HA titer of 1:1,024 (Figure 2E). This result correlates with the electron microscopy and immunoassays described above.

### Role of Temperature in Regulation of *fimA*

**Figure 3:**
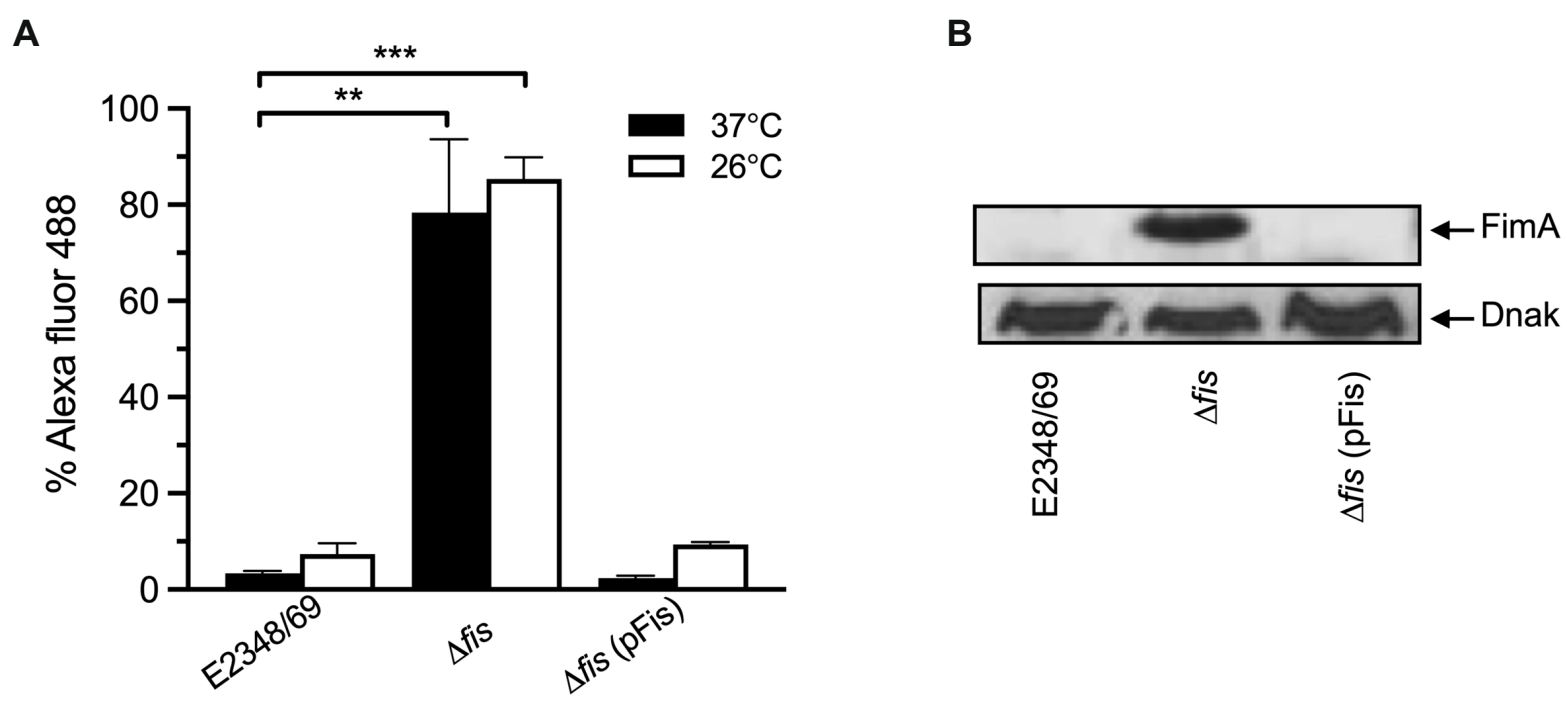
Complementation of E2348/69Δ*fis* with *fis* on a plasmid restores negative regulation. **(A)** Flow cytometry and **(B)** Western blot to determine production of T1P by wild-type E2348/69, E2348/69Δ*fis* mutant, and E2348/69Δ*fis* complemented with *fis* on pUC19. These data are the mean of at least three experiments performed in triplicate on different days. **p < 0.01; ***p < 0.001.

In most laboratory *E. coli* strains the production of T1P is favored by growth in static liquid cultures at 26°C. We wanted to know if temperature had a role in the regulation of T1P in EPEC. Thus, we compared T1P production and transcription levels of *fimA* in the E2348/69 wild-type strain, the Δ*fis* mutant, and the Δ*fis* mutant complemented with *fis* on a plasmid. The data show that T1P production in the Δ*fis* mutant is increased 68-fold and 16-fold at 37°C and 26°C, respectively, with respect to the wild-type strain. E2348/69 produced extremely low levels of T1P at either temperature. Complementation of the Δ*fis* mutant with *fis* on pUC19 (Figure 3A) or low-copy plasmid pBR322 (data not shown) resulted in the absence of T1P as shown for the wild-type strain at both temperatures. These results are in line with the *fimA* expression data obtained by RT-PCR (Figure 3B).

### The Orientation of the *fimS* Switch Correlates With the Strain Phenotype

**Figure 4:**
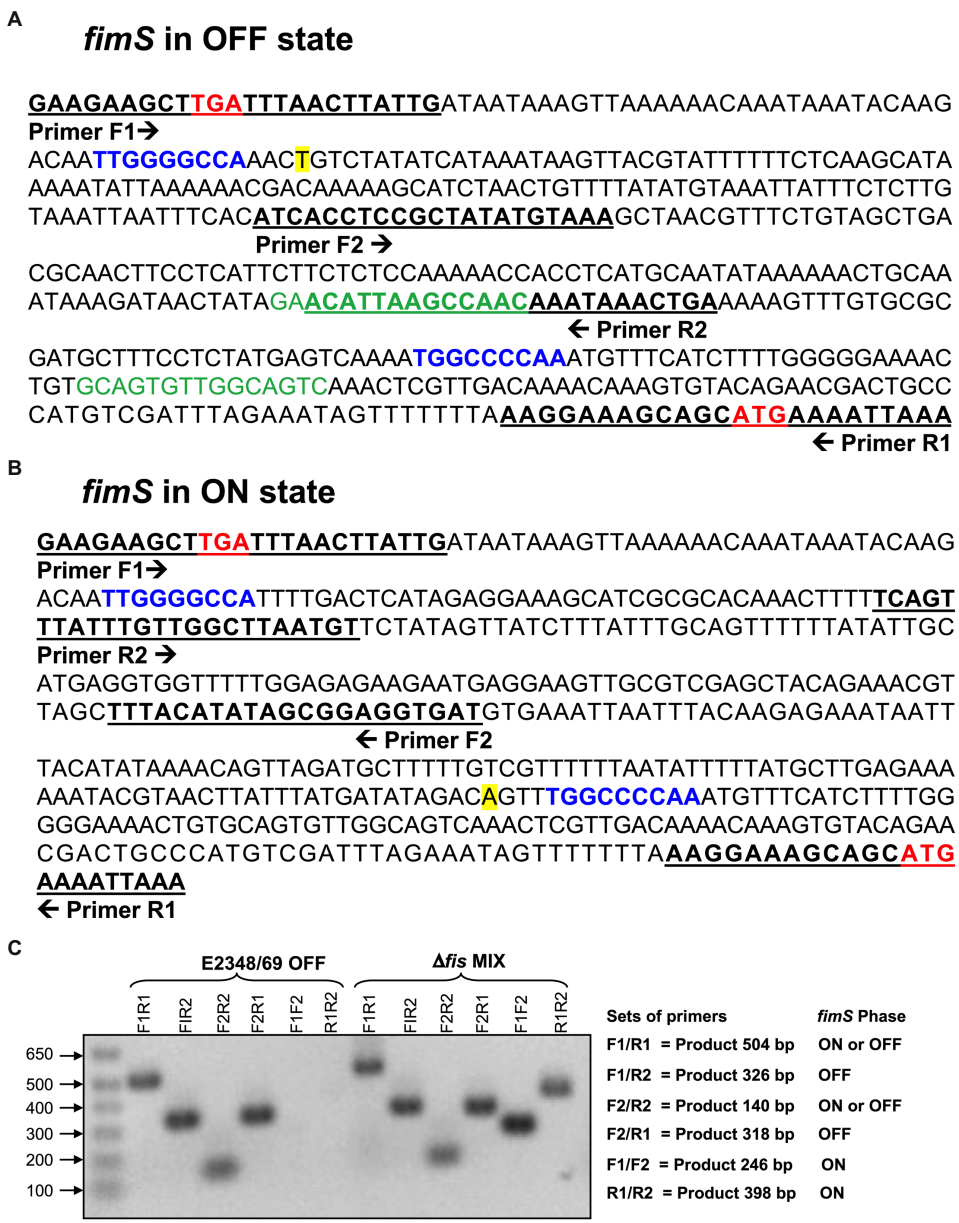
Orientation of the *fimS* invertible element in EPEC strains. **(A)** The nucleotide sequence of the region upstream of the *fimA* structural gene in E2348/69 (Abraham et al., 1985; Klemm, 1986) contains the *fimS* invertible element locked in the OFF orientation. Putative Fis-binding sites are denoted in green. **(B)** Sequence of the *fimS* switch oriented in the ON position. The stop codon (TGA) of *fimE* and the start codon (ATG) of *fimA* are denoted in bold red. The inverted repeat (IR) sequences, IRL (left) and IRR (right), are indicated in blue. The transcription start [A] of *the fimA* promoter is highlighted in yellow. The underlined nucleotide sequences show the binding sites of the primers used in the PCR reaction and the arrows indicate the direction of each primer. **(C)** PCR with different sets of primers show that the Δ*fis* mutant is a mixture of cells containing the *fimS* invertible element oriented in either ON or OFF position. In contrast, the *fimS* switch in the E2348/69 is oriented in the OFF position. The set of primers used, products expected, and the *fimS* phase for each set of primers used are shown on the right.

The *fimS* switch contains a promoter that directs transcription of the *fimA* subunit gene in one orientation (ON), but not in the other (OFF) orientation (McClain et al., 1991). To learn about the orientation of the *fimS* switch in E2348/69 and the Δ*fis* mutant we used PCR with different sets of forward and reverse primers derived from different regions of the *fimS* switch. Amplicons of the expected sizes were obtained in all but two of the combinations of primers in E2348/69; no amplicons were obtained with F1F2 and R1R2 primers confirming that the *fimS* switch in the wild-type strain E2348/69 is locked in the OFF orientation. In contrast, the *fis* mutant showed a mixed population of variants containing the *fimS* invertible element in the ON and OFF orientations as determined by the size of the PCR products that were obtained with all the combinations of primers tested (Figures 4A–C).

### Role of Fis in the Transcriptional Expression of the *fimA, fimB*, and *fimE* Promoters

**Figure 5:**
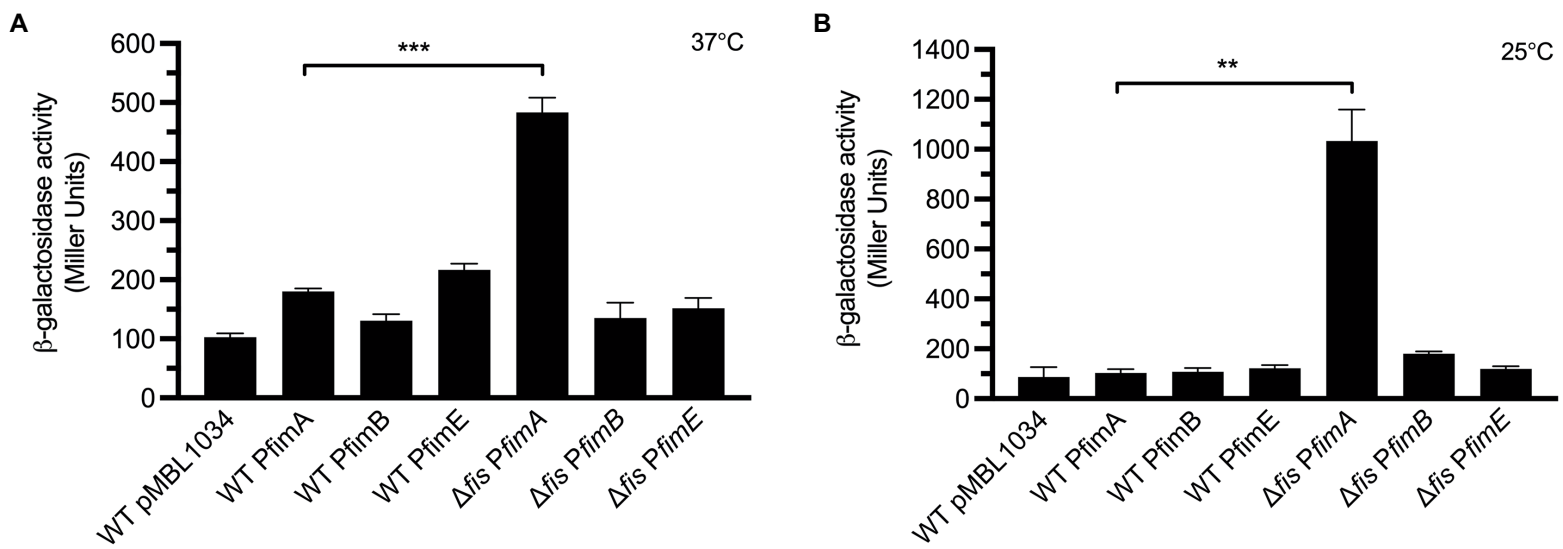
Analysis of transcriptional expression. Fusion constructs consisting of *fimA*, *fimB*, and *fimE* promoters fused to the promoter-less *β*-galactosidase reporter gene were employed to quantitatively determine the expression of *fim* promoters. **(A,B)** A 3-fold and 10-fold increased activity of the P*fimA* promoter in the E2348/69Δ*fis* was recorded at 37°C and 25°C, respectively, in comparison with the wild-type strain. No transcriptional activity was seen in the rest of the fusions. E2348/69 and E2348/69Δ*fis* carrying pMLB1034 were used as negative controls. These data are the mean of at least three experiments performed in triplicate on different days. **p < 0.01; ***p < 0.001.

We wanted to elucidate if the negative effect on the expression of T1P by Fis was at the level of transcription of the fimbrial subunit gene *fimA* or the recombinases *fimB* and *fimE*. For this purpose, transcriptional fusions containing the promoter regions of *fimA*, *fimB*, and *fim*E were fused to the promoter-less *β*-galactosidase reporter gene in plasmid pMBL1034. The resulting fusions were sequenced for confirmation and the plasmids mobilized into EPEC E2348/69 and the Δ*fis* mutant. Transcriptional analysis of these fusions was performed with the strains growing at 25°C and 37°C in LB broth. E2348/69 carrying the *β*-galactosidase reporter gene alone in plasmid pMLB1034 was used as negative control. While a 4.7-fold (Figure 5A) and 10-fold increase in transcription activity of the *fimA* promoter (P*fimA*) was obtained in E2348/69Δ*fis* at 37°C and 25°C, respectively, with respect to the wild-type strain carrying the pMLB1034, no significant transcriptional activity was seen in the strains carrying the P*fimB* or P*fimE* fusions (Figure 5B). It is clear from these experiments that in the absence of Fis, particularly at 25°C, the inversion of the *fimS* switch is favored to the ON orientation allowing transcription from the P*fimA* only and yielding increased production of T1P. This result suggests that the negative effect of Fis is not at the level of transcription of any of the recombinases but most likely due to the binding of Fis to P*fimA* region, cooperating with FimE in locking the *fimS* switch to the OFF position.

### FimE Recombinase Requires Fis to Efficiently Invert the *fimS* Switch to the OFF State

**Figure 6:**
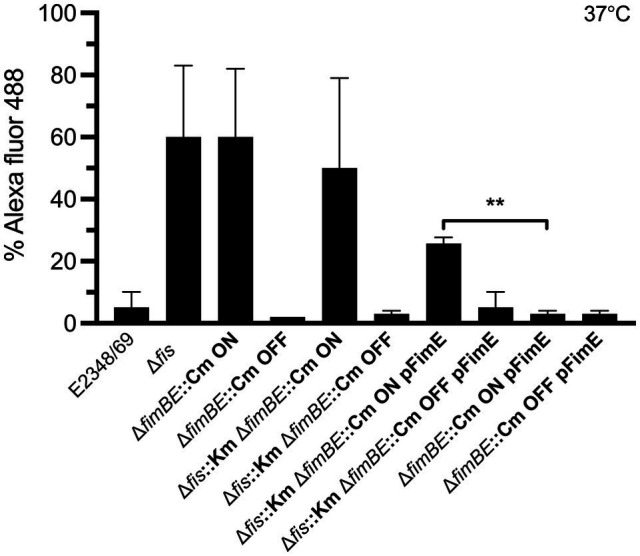
FimE recombinase requires Fis to efficiently switch the *fimS* to the OFF state. T1P expression analysis using double and triple mutants in *fimB*, *fimE*, and *fis* with the *fimS* switch locked in either ON or OFF orientation and complemented with pFimE to evaluate T1P production. T1P was detected by flow cytometry using specific rabbit antibodies anti-T1P and goat anti-rabbit IgG Alexa Fluor conjugate. The Δ*fis*, Δ*fimBE* ON, and Δ*fis* Δ*fimBE* ON strains with the *fimS* locked in the ON orientation produce high levels of T1P pili. Although the E2348/69 Δ*fis* strain has a mix of the *fimS* orientations. No T1P expression was detected in the Δ*fimBE*::Cm and Δ*fis*::Km Δ*fimBE*::Cm strains with the *fimS* locked in the OFF orientation. Interestingly, a 20-fold expression of T1P was displayed in the Δ*fis*::Km Δ*fimBE*::Cm ON strain (*fimS* locked in the ON orientation) complemented with pFimE plasmid as compared to the Δ*fimBE*::Cm ON pFimE strain (*fimS* locked in the ON orientation), which was able to switch the *fimS* element to the OFF state completely. These data are the mean of at least three experiments performed in triplicate on different days. **p < 0.01.

Next, we wanted to determine if the mechanism of inversion of the *fimS* switch required Fis. Our hypothesis was that the FimE recombinase requires Fis to efficiently invert the *fimS* switch to the OFF state. The data obtained so far indicated that in the absence of Fis, high levels of *fimA* expression are displayed. We inquired if this result was caused by a shift of the *fimS* switch as a consequence of the absence of Fis. Thus, we constructed a set of double and triple mutants of *fimE, fimB*, and *fis* containing the *fimS* switch locked in the ON or OFF orientation. The mutants were then complemented with *fimE* on a plasmid. The strains generated were grown overnight in LB medium at 37°C and processed for flow cytometry using anti-T1P antibodies (Figure 6). Similar levels of T1P production were found in the Δ*fis* mutant, the double Δ*fimBE*, and triple Δ*fisfimBE* mutants in which the *fimS* switch is locked in the ON orientation (Figure 6). Interestingly, when the *ΔfimBE*-ON strain was complemented with pFimE, the production of T1P returned to wild-type levels. However, the Δ*fisfimBfimE* strain in the same orientation complemented with pFimE showed 2-fold reduction in T1P production with respect to the triple mutant, but yet, expressed 5 times more T1P than the wild-type strain, suggesting that FimE-mediated inversion of the *fimS* switch to the OFF state requires Fis.

## Discussion

T1P are the most common and best-characterized fimbrial adhesins in the *Enterobacteriaceae* family. While T1P is well-recognized as a virulence factor in the pathogenesis scheme of UPEC and APEC, the role of T1P in the colonization of the human gut mucosa by EPEC is uncertain. Production of T1P is transcriptionally regulated by phase variation, a mechanism that involves the inversion of the *fimS* switch located immediately upstream of *fimA* (Abraham et al., 1985; Klemm, 1986) allowing for two different orientations (ON and OFF). The phase variation of T1P allows a population of bacteria to generate a number of phenotypic variants, some of which may be better suited to colonize certain host niches, for example UPEC expresses T1P in the bladder where it can bind the mannose-rich uroplakin receptors (Thumbikat et al., 2009). As the bacteria ascend to the kidneys the pH drops and the osmolarity increases, which trigger H-NS, RpoS, and OmpR regulators to directly or indirectly shut down *fimB* and *fimE* expression and to lock the *fimS* element in the phase-OFF position (Schwan, 2011). Therefore, the expression of *fim* genes is most optimal during stationary growth phase, in liquid broth, at temperatures below 37°C, and under low osmolarity. It is clear then that expression of T1P is tightly controlled by regulatory genes that determine whether the bacteria will produce or not T1P during interaction with the host or under *in vitro* conditions. Many global regulators, such as H-NS, integration host factor (IHF), RpoS, leucine-responsive regulatory protein (Lrp), CRP-cAMP, known to be involved in regulation of metabolism, stress response, or production of virulence factors, have also been shown to affect T1P expression in response to growth conditions (Martínez-Antonio et al., 2008; Bessaiah et al., 2022). In AIEC, Fis represses expression of *fimE*, and consequently, the *fimS* switch is oriented in the OFF position (Miquel et al., 2010). The role of Fis in the regulation of T1P in EPEC is so far unknown.

In the present study, we inquired about the reasons why E2348/69 lacks T1P although it contains an intact T1P operon. We began by asking if any of the best-known global and virulence regulators described in EPEC played a role in the negative regulation of T1P. E2348/69 isogenic mutants in *perABC, ler, grlA*, and *grlR* (virulence regulators) as well as in *hns, ihf, rpoS, qseA*, and *fis* (global regulators) were tested for the production of T1P. To our surprise, only the Δ*fis* mutant showed a significant increase of FimA synthesis and T1P production, strongly suggesting that Fis acts as a negative regulator of *fimA* expression.

This led us to hypothesize that in wild-type conditions Fis is involved in maintaining the phase variation *fimS* switch oriented in the OFF position and probably is acting in concert with the FimE recombinase to repress *fimA* expression. This is confirmed by the high levels of *fimA* expression found in the Δ*fis* mutant Interestingly, we did not find downregulation of *fimE* occurring in the *fis*-negative mutant. This is in contrast to a published report on AIEC strain LF82 that showed that a LF82Δ*fis* mutant exhibited upregulation of *fimE* indicating that Fis promotes orientation of the *fimS* switch in the OFF state by downregulating the expression of the FimE recombinase (Miquel et al., 2010). These data suggest that the regulation exerted over the *fim* operon by the Fis-FimE couple occurs in various ways in different pathogenic *E. coli* strains.

**Figure 7:**
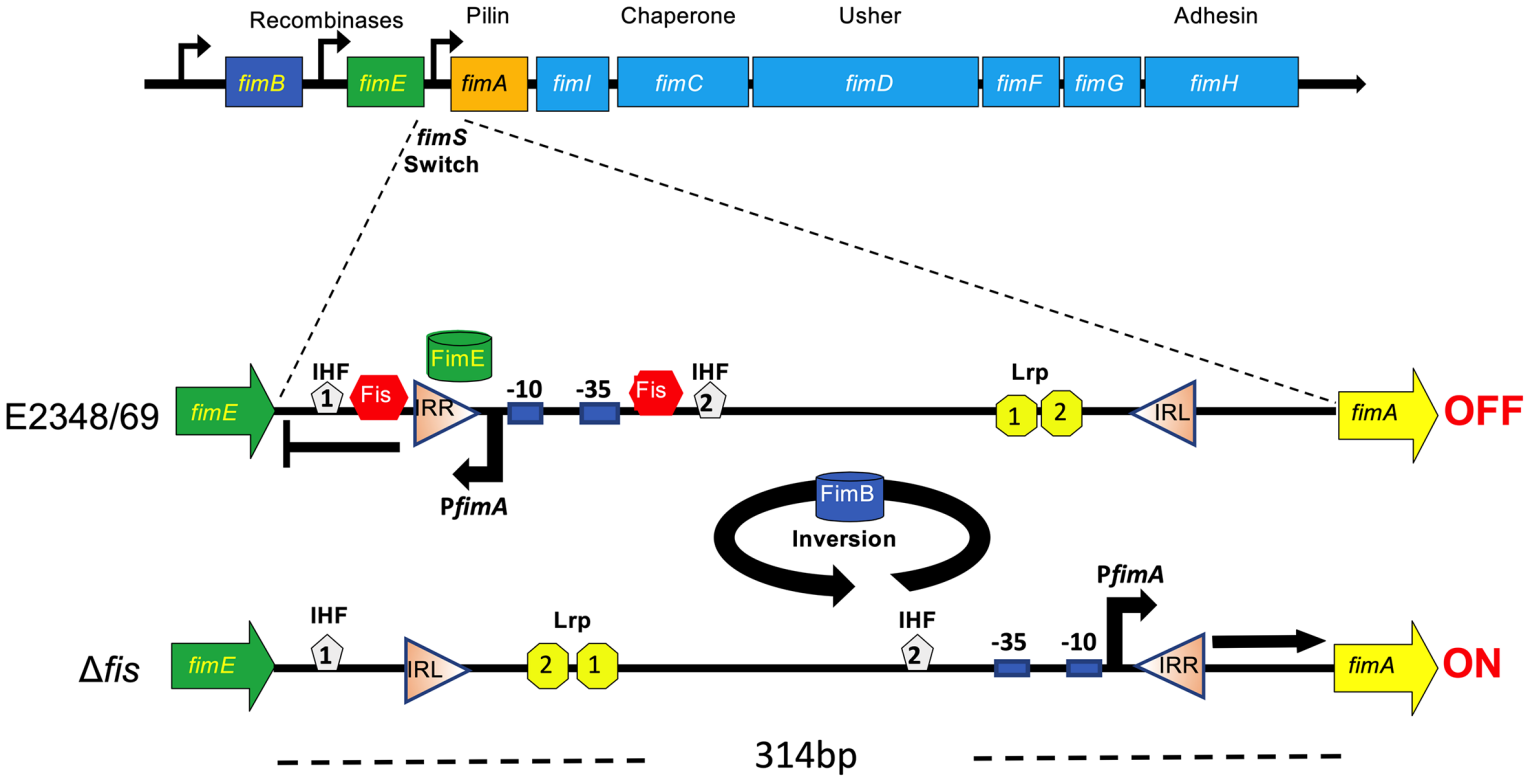
Model for the role of Fis in the site-specific inversion of the *fimS* switch in EPEC. The orientation of the *fimS* invertible segment (314 bp long) located between *fimE* and *fimA*, is controlled by two recombinases, FimB and FimE. FimE locks the *fimS* switch in the OFF orientation while FimB inverts the *fimS* switch in both directions. The *fimS* switch is flanked by the left and right inverted repeats (IRL and IRR, respectively). In EPEC E2348/69, the inversion of the *fimS* to the OFF position is mediated by FimE and Fis. Based on the data obtained, we postulate that in the absence of Fis (e.g., ∆*fis* mutant), high levels of *fimA* expression are displayed and that FimE-mediated inversion of the *fimS* switch to the OFF state requires Fis. The putative binding sites of IHF, Lrp and Fis shown are based on the nucleotide sequence homology of the *fimS* switch between different *Escherichia coli* pathotypes.

The *fimS* switch contains a promoter that directs the transcription of the *fimA* subunit gene in the ON orientation but not in the other (McClain et al., 1991). An important question to address was to determine the orientation of the *fimS* switch in E2348/69 to explain why T1P production is on the OFF state and to inquire about the role of Fis in this event. Thus, using different set of primers derived from different regions of the *fimS* switch we compared amplificons obtained in the wild-type and the Δ*fis* mutant. The analysis of the amplicons confirmed that the *fimS* switch in the wild-type strain E2348/69 is locked in the OFF orientation while the *fis* mutant displayed a mixed population of variants containing the *fimS* invertible element in the ON and OFF orientations. It is tempting to speculate that having a mix population of bacterial cells in the ON and OFF states would be of benefit for attachment to intestinal mannose receptors or for detachment from the gut to exit and colonize other hosts, respectively. The data indicate that the presence of Fis ensures the OFF orientation of the *fimS* switch and therefore we hypothesized that perhaps Fis does this by regulating the expression of *fimA* or the *fimB* and *fimE* recombinase genes (Figure 7). Using promoter-less β-galactosidase transcriptional fusions containing the promoter regions of *fimA*, *fimB*, and *fim*E we determined transcription levels of these genes in E2348/69 and the Δ*fis* mutant growing at 25°C and 37°C in LB broth. Except for the P*fimA* whose expression increased in the Δ*fis* mutant at both temperatures, no significant transcriptional activity was seen in the strains carrying the P*fimB* or P*fimE* fusions. Thus, in the absence of Fis, particularly at 25°C, the *fimS* switch is oriented in the ON position yielding increased *fimA* expression and production of T1P. In contrast, when Fis is present, T1P expression is repressed likely due to the binding of Fis to the *fimS* element and together with FimE, they lock the *fimS* switch on the OFF orientation. To confirm this, we constructed double and triple mutants *fimE*, *fimB*, and *fis* containing the *fimS* locked in the ON or OFF orientation and complemented the mutants with *fimE* on a plasmid (pFimE). T1P production was similar in the Δ*fis*, the double Δ*fimBE* and triple Δ*fisfimBE* mutants with the *fimS* switch locked in the ON orientation. Notably, the Δ*fimBE*-ON(pFimE) strain displayed wild-type levels of T1P while the Δ*fisfimBE*-ON(pFimE) strain expressed 5 times more T1P than the wild-type strain. In all, these data strongly suggest that the FimE-mediated inversion of the *fimS* switch to the OFF state requires Fis. It is possible that Fis stimulates site-specific DNA recombination in conjunction with FimE.

The *E. coli* Fis protein regulates a diverse set of reactions including recombination, transcription, and replication (Finkel and Johnson, 1993) and it does this by binding to specific promoter DNA sequences whose base composition varies enormously. In this study, we sought to investigate the presence of Fis-binding sites within the *fimS* invertible element. Analysis of the promoter of *fimA* in OFF orientation shows two predicted Fis-binding sites (Figure 5A), which correlate with the consensus Fis-binding sequence previously reported (Finkel and Johnson, 1993; Hengen et al., 1997; Schneider et al., 2001; Feldman et al., 2006). Future protein–DNA binding will help to understand the interaction of Fis with nucleotide sequences within the *fimS* switch in EPEC. Fis levels in *E. coli* vary greatly during the course of growth being elevated in early exponential phase and undetectable after stationary phase upon a nutrient up-shift and in response to changing nutritional conditions and this variation may be important for its physiological roles in the cell (Ball et al., 1992; Nilsson et al., 1992; Ali Azam et al., 1999). The fact that T1P are mainly produced during stationary growth phase when Fis levels are low, is in line with our finding that Fis acts as a negative regulator of T1P expression in EPEC. In contrast to Fis, intracellular levels of H-NS are generally high and quite constant, (Ball et al., 1992; Dillon and Dorman, 2010). Published data show that many promoters regulated by Fis are also regulated by H-NS (Dorman, 2007), from which we could speculate that during early exponential growth, Fis and H-NS repress the expression of the *fim* operon. However, here we found that in contrast to the ∆*fis* mutant that expressed high levels of *fimA* and abundant T1P, the ∆*hns* mutant expressed low levels of *fimA* but showed no detectable T1P. It is possible that the lack of Fis has an indirect effect on the expression or function of other transcriptional factors, such as *ihf* or *rpoS* or virulence regulators. Nevertheless, in contrast to what has been reported in UPEC (Bessaiah et al., 2022), the EPEC ∆*ihf* and ∆*rpoS* mutants did not show *fimA* expression, FimA synthesis, or T1P production. Likewise, the mutants in virulence regulators showed no-to-negligible amounts of T1P. From these experiments, we conclude that Fis is required for the FimE-mediated ON-to-OFF switching. In all, this study reveals that the regulation of T1P in different *E. coli* pathotypes depends of a complex network of regulatory elements that work in concert to facilitate tropism and colonization of the appropriate niches in the host.

## Experimental Procedures

### Strains and Culture Conditions

Bacterial strains used in this study are listed in Supplementary Table 1 and were grown on Luria-Bertani (LB) at 37°C, unless otherwise noted. When necessary, kanamycin or ampicillin was added at a concentration of 50 or 100 μg/ml, respectively.

### Construction of Isogenic Mutants

Non-polar deletion mutants in *fis*, *fimB*, and *fimE* genes were generated by the lambda Red recombinase method previously described (Datsenko and Wanner, 2000). The primers employed for DNA amplification are listed in Supplementary Table 2. Primers *fis*/H1P1 and *fis*/H2P2 were employed to mutate *fis* in EPEC E2348/69. Primers G356 and G357 and primers G360 and G361 were employed to mutate *fimB* and *fimE*, respectively, in EPEC E2348/69. Primers flanking the *fis*, *fimB*, and *fimE* genes as well as primers inside the kanamycin and chloramphenicol resistance gene were used to confirm the required gene replacement by PCR (Supplementary Table 2). To complement the *fis* mutation, the pFis vector carrying the *fis* gene was used (Saldaña et al., 2009). To generate the deletion of both recombinase genes (*fimB and fimE*) we used primers G356 and G361.

### SDS-PAGE and Immunoblotting

The bacterial suspensions were adjusted to an absorbance of 0.7 at the optical density (OD) at 600 nm (OD_600_). Equal numbers of bacteria were used to prepare whole cell extracts. To dissociate T1P from the bacteria, the cultures were treated with acidified water (pH 1.8), boiled for 10 min in denaturation sample buffer, neutralized to pH 7.2, and then resolved by SDS-PAGE and transferred to PVDF membranes. The membranes were incubated with rabbit anti-T1P (1:3,000) in PBS-Tween 80 for 1 h followed by anti-rabbit IgG-peroxidase conjugate (Sigma; 1:5,000) and the reaction was visualized by addition of a chemiluminescent substrate (Amersham). Anti-DnaK antibody was used to control for the amount of protein loaded in the gels.

### Flow Cytometry

Flow cytometry was used to quantitate the production of T1P by the EPEC strains. These strains were grown overnight in LB media at 37°C or 26°C and the optical density adjusted to an OD_600_ of 1.1. Forty-five μl aliquots were incubated for 1 h on ice with 25 μl of anti-T1P antibodies at a dilution of 1:500. After three gentle washes with PBS, the bacteria were resuspended in 25 μl of a 1:500 dilution of goat anti-rabbit IgG (H + L) Alexa Fluor conjugate (Invitrogen, Carlsbad, CA). After 1 h incubation at 4°C, the bacteria were gently washed three times with PBS and resuspended in 800-μl final volume of PBS. For the analysis, the bacteria were labeled with 3 μl of a propidium iodide solution (Sigma, St. Louis, MO). Propidium iodide (red) was visualized through a 42 nm band pass centered at 585. These experiments were repeated in triplicate. The FITC (green) fluorescence emission was collected through a 30 nm band pass filter centered at 530 in which 50,000 events were measured. The samples were analyzed at the ARL Biotechnology/ACCC Cytometry Core Facility at the University of Arizona, by using a FACScan (Becton Dickinson, Franklin Lakes, NJ).

## RT-PCR

Total RNA was extracted from LB-grown bacterial cultures using TRIzol Reagent (Invitrogen) following the manufacturer’s guidelines. Prior to RT-PCR, 2 μg of total RNA were treated with RQ1 RNAse-free DNase, according to the manufacturer’s protocol. Specific transcripts were amplified using the one-step RT-PCR kit (Qiagen) and 0.1 mg/ml of total RNA as template. 16S RNA (*rrsB*) was used as a loading control.

### Ultrastructural Studies

The pili on the bacterial surface were visualized by negative staining with 1% phosphotungstic acid followed by transmission electron microscopy (TEM). For immunogold labeling, samples were incubated with 1:10 dilution of rabbit anti-T1P antibody in normal horse serum for 1 h followed by incubation with anti-rabbit IgG conjugated to 10 nm gold particles (1:10) for 1 h. After washing the grids were stained as before (Girón et al., 2002).

### Purification of Pili

Pili produced by E2348/69Δ*fis* were purified from the bacteria cultivated in 40 plates (150 × 15 mm) of LB agar and resuspended in 100 ml of distilled water. The suspension was vigorously shaken for 5 min to shear the pili from the bacterial cells. The supernatant was obtained by centrifugation at 10,408 x *g* for 20 min in a Sorval GSA rotor. A second centrifugation step at 17,210 x *g* for 20 min in a Sorval SS34 rotor was performed to remove bacterial debris. The clear supernatant was spun at 148,230 x *g* for 3 h in a Beckman 70 Ti rotor to concentrate the pili. The pili was resuspended in 0.1 mM Tris–HCl and centrifuged 18 h at 256,136 x *g* in a Beckman SW 40 Ti rotor in a Beckman L-100 K ultracentrifuge using a cesium chloride/1% sarkosyl gradient to obtain purified fimbriae (Tacket et al., 1987).

### Production of Rabbit Polyclonal Antiserum against T1P

The purified T1P was used to custom-order polyclonal antibodies at Lampire Laboratories in a New Zealand rabbit by intramuscular injection with Freund’s complete adjuvant at day 0 followed by a second immunization at day 15 in incomplete Freund’s adjuvant. The final bleed was obtained at day 50 and the serum stored at –70°C.

### Hemagglutination Assays

Guinea pig red blood cells (RBC; Lampire, PA) were assayed for agglutination by EPEC and isogenic mutant strains as previously described (Erdem et al., 2007). Hemagglutination (HA) assays were performed with 96-well, round-bottom microtiter plates. Bacteria were adjusted to 10^8^ cells per ml in PBS. Two-fold serial dilutions of the bacteria in 100 ml were incubated with 100 ml 1% RBC suspensions and incubated on ice for 2 h. HA titers were recorded when a pellet of RBC was observed in the well containing only RBC in PBS (Erdem et al., 2007).

### Construction of *fimA::lacZ, fimB::lacZ*, and *fimE::lacZ* Transcriptional Fusions

Transcriptional fusions consisting of the EPEC *fimA, fimB*, and *fimE* promoters linked to promoter-less *lacZ* reporter gene were constructed to monitor the expression of *fimA, fimB*, and *fimE*, respectively. The *fimA* promoter was amplified from E2348/69 using the primers G307 and G135 and cloned into the *EcoRI* and *BamHI* sites in pMLB1034 vector, yielding p*fimA*. To clone the *fimB* promoter (located 610 bp upstream of the start codon) we used primers G310 and G311; primers G308 and G311 were used to clone the *fimE* promoter into the *EcoRI* and *BamHI* sites in pMLB1034 vector, yielding p*fimB* and p*fimE*, respectively. These plasmids were transformed into E2348/69 and E2348/69Δ*fis*, and transcriptional activity of *fimA*, *fimB*, and *fimE* was monitored by measuring *β*-galactosidase activity as previously described (Miller, 1972). As negative control, E2348/69 carrying pMLB1034 was employed.

### *β*-Galactosidase Assays

Transcriptional expression analysis using several fusion constructs (promoters from *fimA*, *fimB*, and *fimE* fused to the *β*-galactosidase reporter gene) were grown with shaking for 21 h at 37°C. We chose this time point to measure *fimA, fimB*, and *fimE* expression because *fim* genes are expressed at stationary phase and Fis is a repressor of stationary phase genes. The cultures were diluted 1:50 in fresh LB and cultivated at 37°C to an OD_600_ of 0.65–0.70. When required the cultures were grown at 37°C or 25°C. The cultures were then diluted 1:5 in Z buffer (0.06 M Na_2_HPO_4_, 0.04 M Na_2_HPO_4_, 0.01 M KCl, 0.001 M MgSO_4_, and 0.05 M *β*-mercaptoethanol) and the *β*-galactosidase activity was assayed using ONPG as a substrate. The color was read in a spectrophotometer (Miller, 1972). The *β*-galactosidase experiments were repeated at least four times in quadruplicate.

### Amplification of the *fimS* Invertible Element in EPEC Strains

Cultures of E2348/69 and the ∆*fis* mutant were analyzed by PCR utilizing sets of different primers (Supplementary Table 2) expanding different regions of the *fimS* switch to determine the orientation of the *fimS* switch in each strain. The absence of amplicons in the E2348/69 strain utilizing sets of primers F1F2 and R1R2 confirm the OFF orientation of the *fimS* switch in this strain. In contrast, the presence of amplicons in the ∆*fis* mutant with these sets of primers confirmed the ON position of the *fimS* switch.

### Statistical Analysis

All data were the averages of at least three independent experiments performed by triplicate. GraphPad Prism 9 software (GraphPad, San Diego, CA, United States) was used for statistical differences. One way ANOVA followed by Tukey’s multiple comparison test and unpaired Student’s *t* test were performed. A *value of p* ≤0.05 was considered statistically significant.

## Data Availability Statement

The original contributions presented in the study are included in the article/Supplementary Material, further inquiries can be directed to the corresponding author.

## Author Contributions

ZS-A and JG conceived and designed the experiments. ZS-A and VM-S performed the experiments. ZS-A, JS-B, JY-S, YM-L, MC-R, JP, and JG analyzed the data. ZS-A, JS-B, and JG wrote the manuscript. All authors contributed to the article and approved the submitted version.

## Conflict of Interest

The authors declare that the research was conducted in the absence of any commercial or financial relationships that could be construed as a potential conflict of interest.

## Publisher’s Note

All claims expressed in this article are solely those of the authors and do not necessarily represent those of their affiliated organizations, or those of the publisher, the editors and the reviewers. Any product that may be evaluated in this article, or claim that may be made by its manufacturer, is not guaranteed or endorsed by the publisher.

## Supplementary Material

The Supplementary Material for this article can be found online at: https://www.frontiersin.org/articles/10.3389/fmicb.2022.882563/full#supplementary-material
