# Supplementary material for: The Fis Nucleoid Protein Negatively Regulates the Phase Variation fimS Switch of the Type 1 Pilus Operon in Enteropathogenic Escherichia coli
Source: Front Microbiol. 2022 Apr 28;13:882563. doi: 10.3389/fmicb.2022.882563 (PMC9096935; doi:10.3389/fmicb.2022.882563)
Supplement: Supplementary file 1 [file Table_1.docx]

**Supplementary Table 1.** *E. coli* strains and plasmids used in this study.

| **Strains** | **Notes** | **Source** |
| --- | --- | --- |
| E2348/69 | EPEC (O127:H6) Wild type | (Levine et al., 1978) |
| E2348/69Δ*fis* | *fis*::km | (Saldaña et al., 2009) |
| E2348/69Δ*fis*(pFis) | *fis*::km complemented with pFis | (Saldaña et al., 2009) |
| E2348/69Δ*fimB* | *fimB*::cm | This study |
| E2348/69Δ*fimE* | *fimE*::cm | This study |
| E2348/69Δ*fis*Δ*fimE* | Double *fis*::km*/fimE*::cm mutant | This study |
| E2348/69Δ*fimBE* with *fimS* in ON | *fimBE*::cm | This study |
| E2348/69Δ*fimBE* with *fimS* in OFF | *fimBE*::cm | This study |
| E2348/69Δ*fis* Δ*fimBE* with *fimS* in ON | *fimBE*::cm *fis*::km | This study |
| E2348/69Δ*fis* Δ*fimBE* with *fimS* in OFF | *fimBE*::cm *fis*::km | This study |
| E2348/69Δ*fimBE* with *fimS* in ON (pFimE) | *fimBE*::cm complemented with pFimE | This study |
| E2348/69Δ*fimBE* with *fimS* in OFF (pFimE) | *fimBE*::cm complemented with pFimE | This study |
| E2348/69Δ*fis* Δ*fimBE* with *fimS* in ON (pFimE) | *fimBE*::cm *fis*::km complemented with pFimE | This study |
| E2348/69Δ*fis* Δ*fimBE* with *fimS* in OFF (pFimE) | *fimBE*::cm *fis*::km complemented with pFimE | This study |
|  |  |  |
|  |  |  |
|  |  |  |
|  |  |  |
| E2348/69(pMLB1034) | Promoter-less control | This study |
| E2348/69(P*fimA*) | *fimA*::*lacZ* transcriptional fusion | This study |
| E2348/69(P*fimB*) | *fimB*::*lacZ* transcriptional fusion | This study |
| E2348/69(P*fimE*) | *fimE*::*lacZ* transcriptional fusion | This study |
| E2348/69Δ*fis*(P*fimA*) | *fimA*::*lacZ* transcriptional fusion | This study |
| E2348/69Δ*fis*(P*fimB*) | *fimB*::*lacZ* transcriptional fusion | This study |
| E2348/69Δ*fis*(P*FimE*) | *fimE*::*lacZ* transcriptional fusion | This study |
| E2348/69∆*grlA* | *grlA*::km mutant | Bustamante et al., 2011 |
| E2348/69∆*grlR* | *grlR*::km mutant | Lara-Ochoa et al., 2021 |
| E2348/69∆*grlRA* | *grlRA*::km mutant | Bustamante et al., unpublished |
| E2348/69∆*ler* | *ler*::km mutant | Bustamante et al., 2011 |
| E2348/69∆*hns* | *hns*::km mutant | Bustamante et al., unpublished |
| E2348/69∆*rpos* | *rpoS*::km mutant | García-Angulo et al., 2012 |
| E2348/69∆*ihf* | *∆himA*::km mutant | García-Angulo et al., 2012 |
| E2348/69∆*perA* | *∆perA*::km mutant | Bustamante et al., 2011 |
| E2348/69∆*perB* | *∆perB*::km mutant | Bustamante et al., 2011 |
| E2348/69∆*perC* | *∆perC*::km mutant | Bustamante et al., 2011 |
| E2348/69∆*qseA* | *∆qseA*::km mutant | This study |
| Plasmids |  |  |
| pKD46 | Red recombinase system plasmid |  |
| pKD4 | Kanamycin cassette template plasmid |  |
| pKD3 | Chloramphenicol cassette template plasmid |  |
| pFis | E2348/69*fis* in pUC19 | (Saldaña et al., 2009) |
| pFis2 | *fis* cloned in pBR322 with its regulatory region | This study |
| pFimE | *fimE* cloned in pBR322 with its regulatory region | This study |
|  |  |  |
| pMLB1034 | Promoter-less *lacZ* | (Uhlich et al., 2002) |
| P*fimA* | Promoter *fimA*::*lacZ* in pMLB1034 | This study |
| P*fimB* | Promoter *fimB*::*lacZ* in pMLB1034 | This study |
| P*fimE* | Promoter *fimE*::*lacZ* in pMLB1034 | This study |
