# Supplementary material for: The Fis Nucleoid Protein Negatively Regulates the Phase Variation fimS Switch of the Type 1 Pilus Operon in Enteropathogenic Escherichia coli
Source: Front Microbiol. 2022 Apr 28;13:882563. doi: 10.3389/fmicb.2022.882563 (PMC9096935; doi:10.3389/fmicb.2022.882563)
Supplement: Supplementary file 2 [file Data_Sheet_1.docx]

**Supplemental References**

1. Bustamante, V. H., Villalba, M. I., García-Angulo, V. A., Vázquez, A., Martínez, L. C., Jiménez, R., et al. (2011). PerC and GrlA independently regulate Ler expression in enteropathogenic *Escherichia coli*. *Mol. Microbiol*. 82:398-415. doi: 10.1111/j.1365-2958.2011.07819.x
2. Lara-Ochoa, C., González-Lara, F., Romero-González, L. E., Jaramillo-Rodríguez, J. B., Vázquez-Arellano, S. I., Medrano-López, A., et al. (2021). The transcriptional activator of the bfp operon in EPEC (PerA) interacts with the RNA polymerase alpha subunit. *Sci Rep*. 11:8541. doi: 10.1038/s41598-021-87586-0
3. Levine, M. M., Bergquist, E. J., Nalin, D. R., Waterman, D. H., Hornick, R. B., Young, C. R. et al. (1978). *Escherichia coli* strains that cause diarrhoea but do not produce heat-labile or heat-stable enterotoxins and are non-invasive. *Lancet*. 1:1119-1122. doi: 10.1016/s0140-6736(78)90299-4
4. Saldaña, Z., Xicohtencatl-Cortes, J., Avelino, F., Phillips, A. D., Kaper, J. B., Puente, J. L., et al. (2009). Synergistic role of curli and cellulose in cell adherence and biofilm formation of attaching and effacing *Escherichia coli* and identification of Fis as a negative regulator of curli. *Environ. Microbiol.* 11:992-1006. doi: 10.1111/j.1462-2920.2008.01824.x
5. García-Angulo, V. A., Martínez-Santos, V. I., Villaseñor, T., Santana, F. J., Huerta-Saquero, A., Martínez, L. C., et al. (2012). A distinct regulatory sequence is essential for the expression of a subset of *nle* genes in attaching and effacing *Escherichia coli*. *J. Bacteriol*. 194:5589-5603. doi: 10.1128/JB.00190-12
6. Uhlich, G. A., Keen, J. E., Elder, R. O. (2002). Variations in the *csgD* promoter of *Escherichia coli* O157:H7 associated with increased virulence in mice and increased invasion of HEp-2 cells. *Infect. Immun*. 70:395-399. doi: 10.1128/IAI.70.1.395-399.2002
