# Supplementary figures and images for: The Fis Nucleoid Protein Negatively Regulates the Phase Variation fimS Switch of the Type 1 Pilus Operon in Enteropathogenic Escherichia coli
Source: Front Microbiol. 2022 Apr 28;13:882563. doi: 10.3389/fmicb.2022.882563 (PMC9096935; doi:10.3389/fmicb.2022.882563)

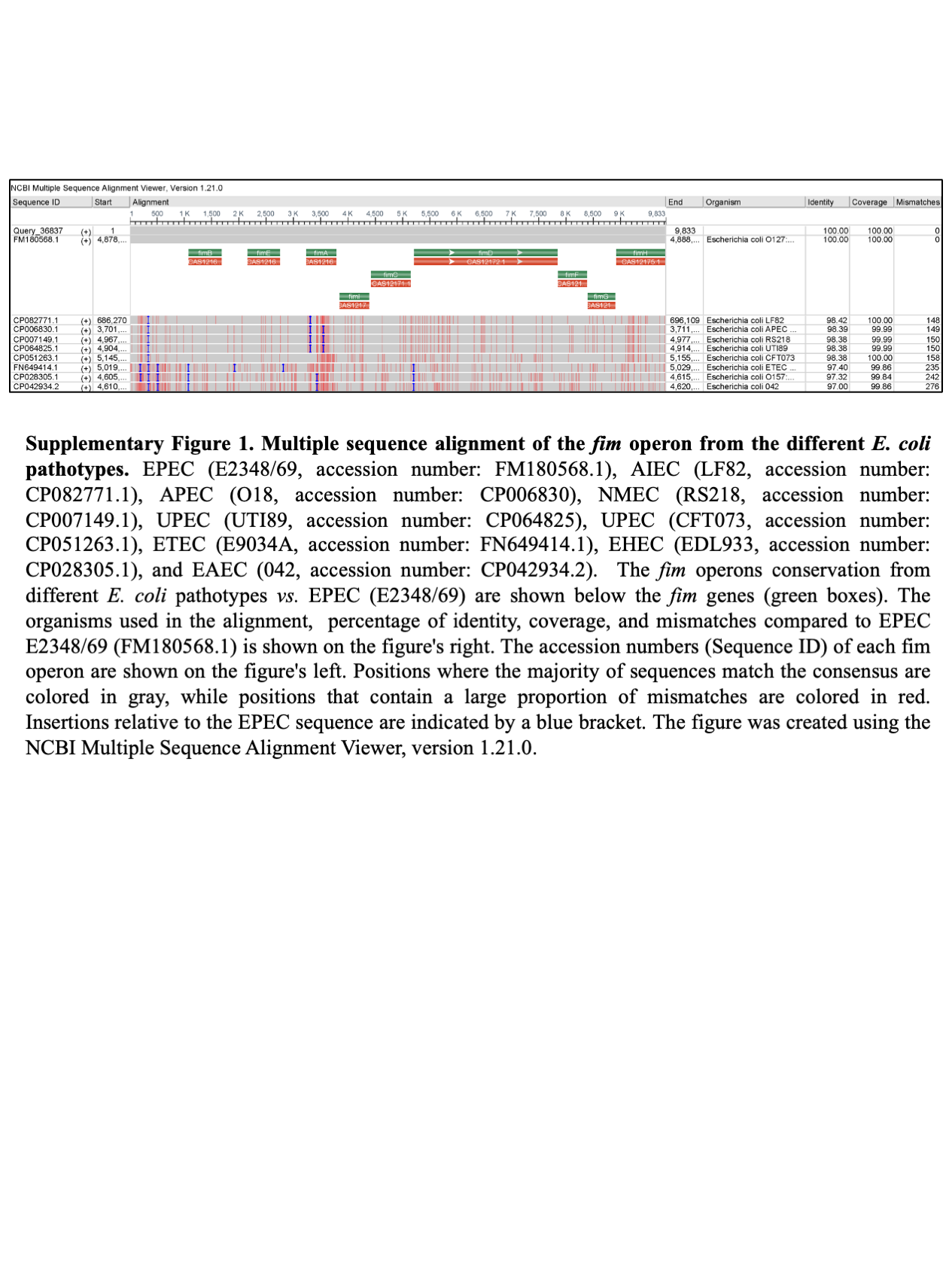

Supplement: Supplementary file 3 [file Image_1.tiff]

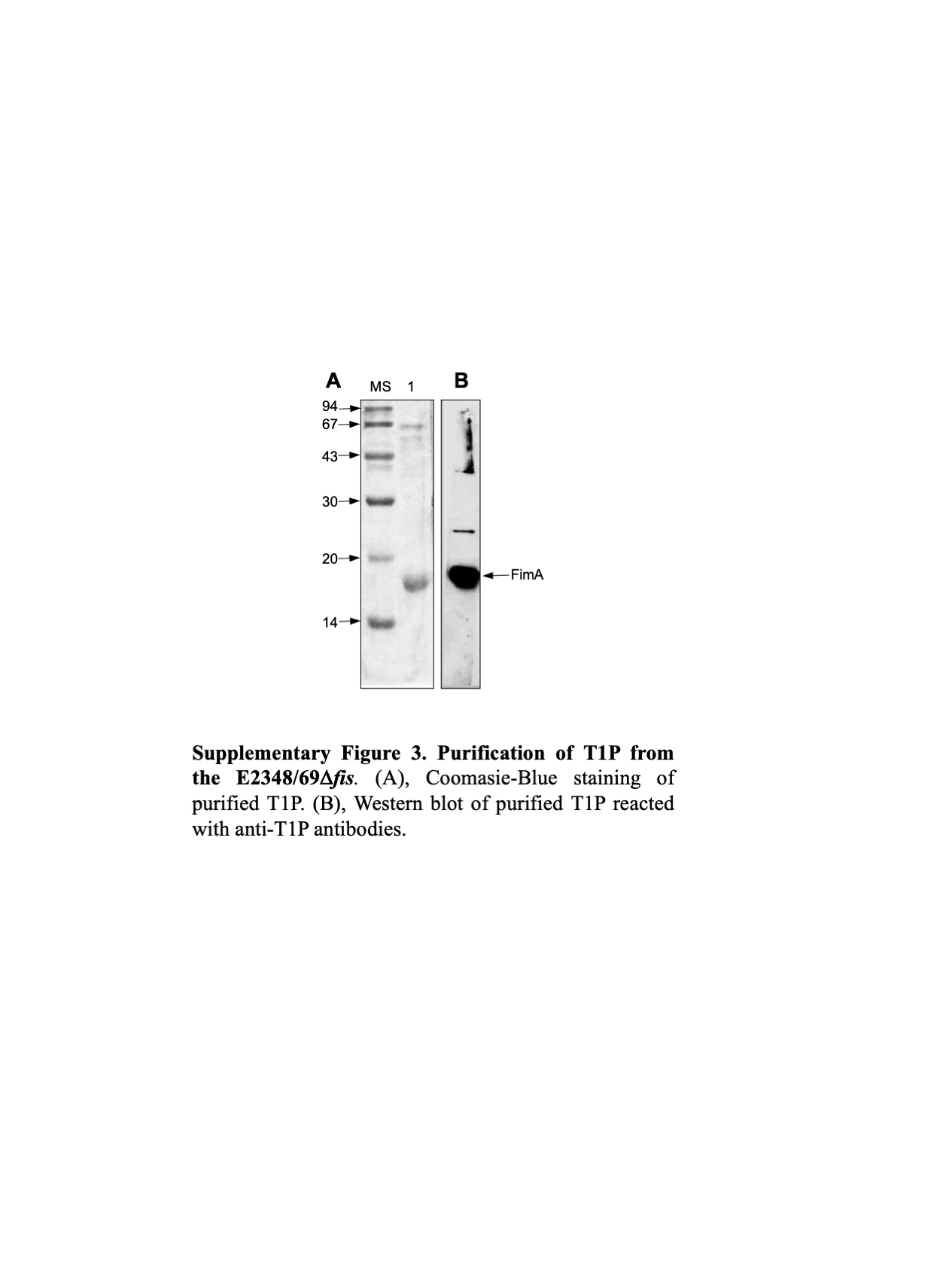

Supplement: Supplementary file 4 [file Image_2.tiff]

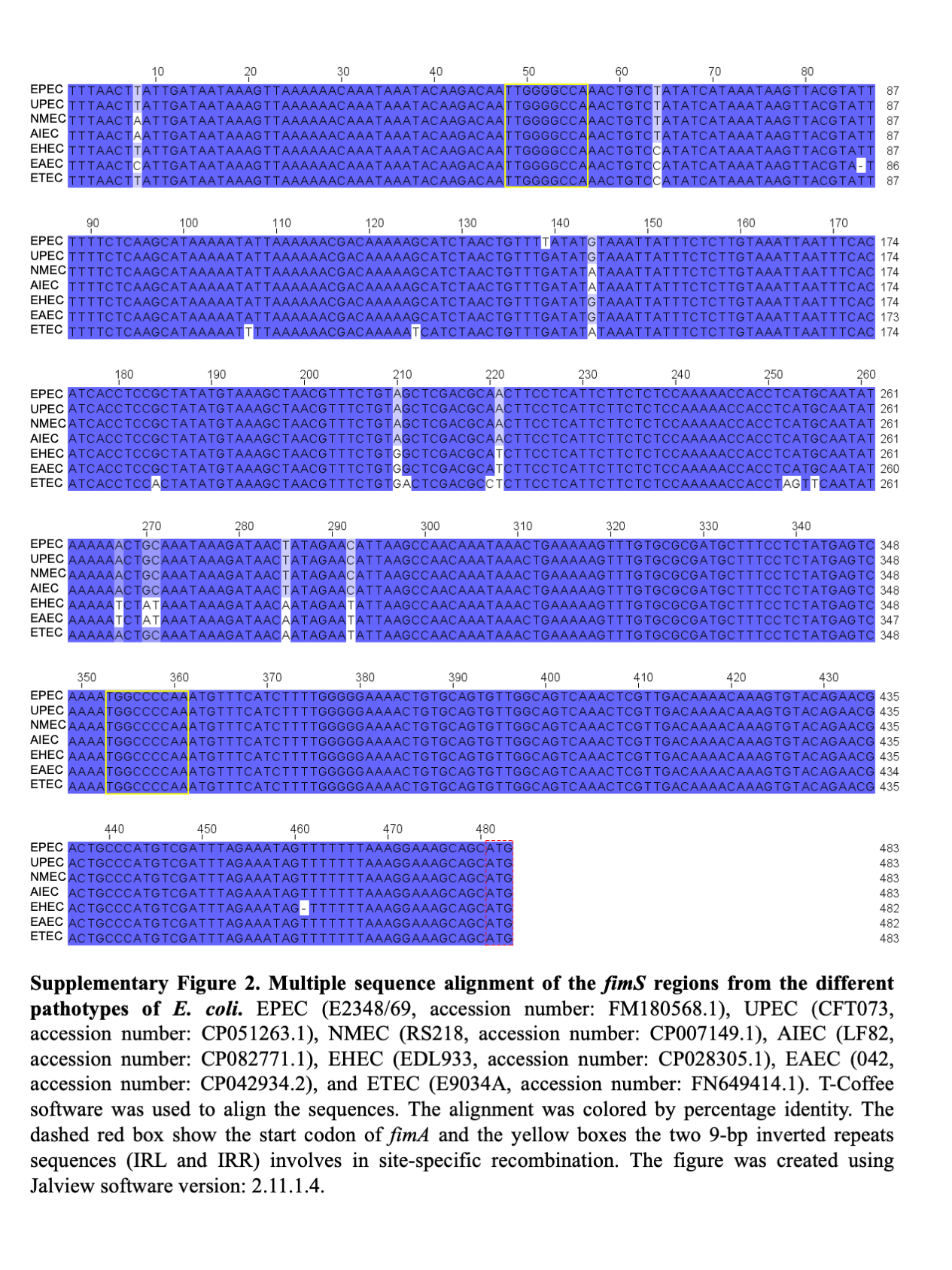

Supplement: Supplementary file 5 [file Image_3.tiff]
